# *Hessebius luculentus*, a new species of the genus *Hessebius* Verhoeff, 1941 from China (Lithobiomorpha, Lithobiidae)

**DOI:** 10.3897/zookeys.741.20061

**Published:** 2018-03-07

**Authors:** Huiqin Ma, Yanmin Lu, Haipeng Liu, Xiaojie Hou, Sujian Pei

**Affiliations:** 1 Scientific Research Office, Hengshui University, Hengshui, Hebei 053000, P. R. China; 2 School of Life Sciences, Hengshui University, Hengshui, Hebei 053000, P. R. China

**Keywords:** China, *Hessebius luculentus* sp. n., Lithobiidae, Qinghai-Tibet Plateau

## Abstract

*Hessebius luculentus* **sp. n.** (Lithobiomorpha: Lithobiidae), recently discovered from Shandan County, Zhangye City, Province Gansu, Qinghai-Tibet Plateau, China, is described. Morphologically it resembles *H. jangtseanus* (Verhoeff, 1942), but can be easily distinguished from the latter by size of Tömösváry’s organ’s, the morphological characters of a protuberance on the dorsal end of the second article of the female gonopods; and obvious differences in the dorsal plectrotaxy of both the 14^th^ and 15^th^ legs. The main morphological characters and a key to the known Chinese species of genus *Hessebius* based on adult specimens are presented.

## Introduction

*Hessebius* was originally proposed as a genus in the family Lithobiidae by Verhoeff (1941) to accommodate the species *H. kosswigi* Verhoeff, 1941 and *H. tauricus* Verhoeff, 1941 described from Turkey. The latter species was reassigned to the genus *Lithobius* Leach, 1814 by Zapparoli (1999). Zalesskaja (1978), Eason (1981), Pei et al. (2010) and Bonato et al. (2011) debated the taxonomic status of *Hessebius* and considered it at generic rank and selected *H. kosswigi* Verhoeff, 1941, from Turkey, as type species by subsequent designation (Verhoeff 1941, Zalesskaja 1978, and Eason 1981). Presently, the genus comprises 12 species (Zapparoli 2016), characterized by the following traits: antennae generally with 20 articles, 13–15 ocelli, forcipular coxosternal teeth 2+2; tergites without posterior triangular projections; legs 14 and 15 thicker than the anterior legs in females, both thicker in males; coxal pores 4–7; the first article of the female gonopods with 2+2 spurs, the second article with a massive expansion and projection on the dorsolateral ridge, and a long claw sometimes with a stout lateral tooth at its base. *Hessebius* has a distribution that extends from Mongolia and south-east China through central Asia (Kazakhstan, Kyrgyzstan, Tajikistan, Turkmenistan), the southern Urals, south-west Russia (Kalmykia and adjacent areas), westwards up to the Middle East (Iran, Armenia) and eastern Mediterranean basin (south-west Turkey, Rhodes, Cyprus, Syria, Palestine, Israel, Jordan, north Egypt, Cyrenaica) (Pei et al. 2010).

The myriapod fauna of China is still poorly known and very little attention has been paid to the study of Lithobiomorpha, with only 74 species/subspecies hitherto known from the country (Ma et al. 2014a, b, 2015; Pei et al. 2010, 2014, 2015, 2016; Qin et al. 2014), among which are three species of *Hessebius* viz., *H. jangtseanus* (Verhoeff, 1942), *H. longispinipes* Ma, Pei & Zhu, 2009 and *H. multiforaminis* Pei, Ma, Zapparoli & Zhu, 2010. In the present study another new species of *Hessebius* from Gansu Province, Qinghai-Tibet Plateau, China, is described and illustrated. The main morphological characters and key to the known Chinese species of genus *Hessebius* are presented.

## Materials and methods

All specimens were hand-collected under leaf litter or stones. The material was examined with the aid of Nikon SMZ-1500 stereomicroscope. The colour description is based on specimens in 75% ethanol, body length is measured from the anterior margin of the cephalic plate to the posterior end of the postpedal tergite. Type specimens are deposited in the School of Life Sciences, Hengshui University, Hengshui, China. Terminology applied to external anatomy follows Bonato et al. (2010).

The following abbreviations are used in the text and tables:

**a** anterior;

**C** coxa;

**F** femur;

**m** median;

**p** posterior;

**P** prefemur;

**S, SS** sternite, sternites;

**T, TT** tergite, tergites;

**Tr** trochanter;

**Ti** tibia.

## Taxonomy

### Lithobiidae Newport, 1844

#### *Hessebius* Verhoeff, 1941

##### Hessebius luculentus sp. n.

Taxon classificationAnimaliaLithobiomorphaLithobiidae

http://zoobank.org/CAA43A06-280A-4127-8BE6-C71C5E7A705A

Figs 1–8

###### Material examined.

**Figures 1–8.**
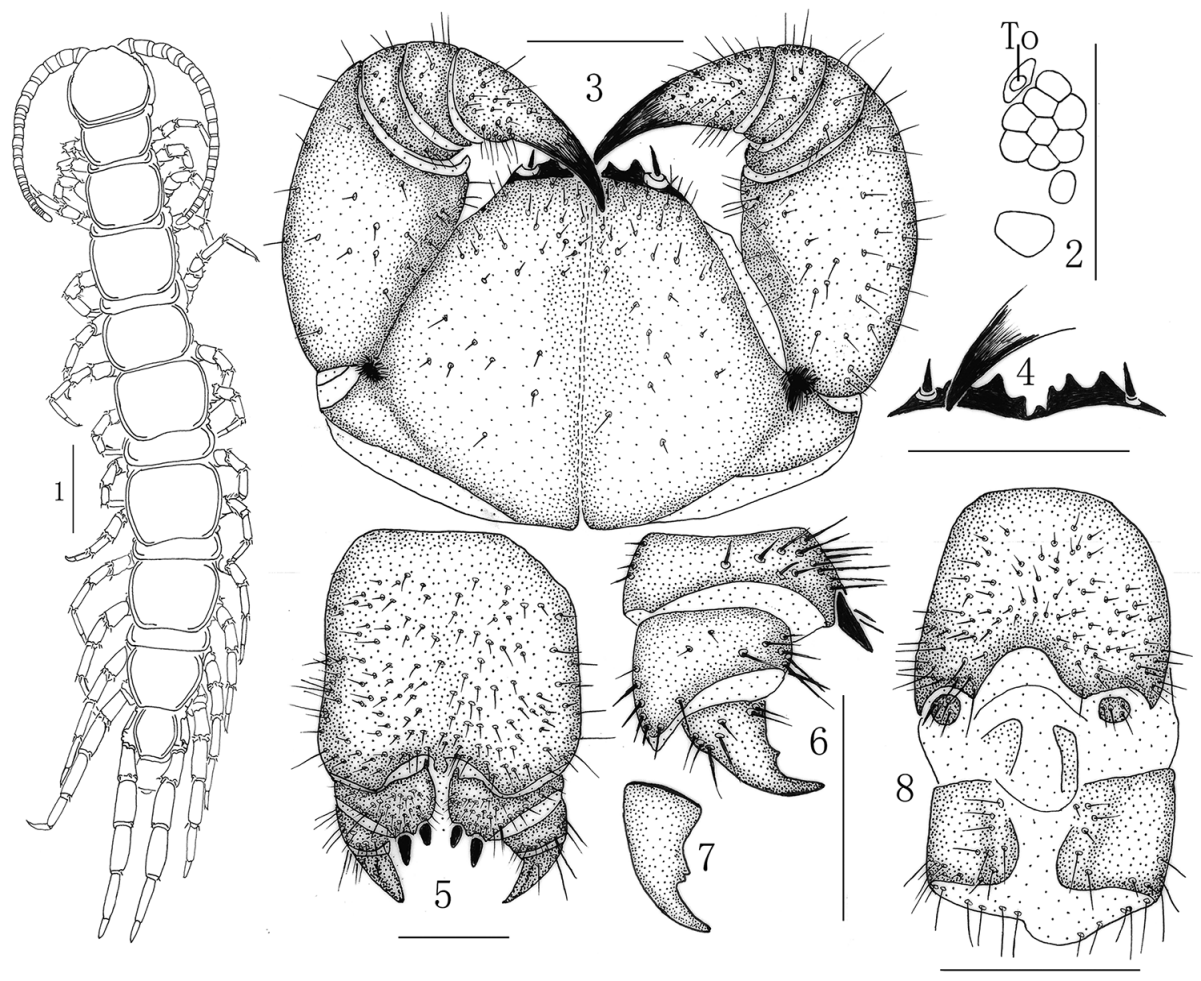
*Hessebius luculentus* sp. n., **1–3** and **8** holotype, male: **1** habitus, dorsal view **2** ocelli and Tömösváry’s organ (**To**), lateral view **3** forcipular coxosternite, ventral view **4–7** paratype, female: **4** anterior margin of forcipular coxosternite, ventral view **5** posterior segments and gonopods, ventral view **6** gonopods, transparent protuberance in dorsolateral view **7** claw of female gonopod, lateral view **8** posterior segments and gonopods, ventral view. Scale bars: 500 μm (**2, 3, 5–8**), 2 mm (**1**); 1 mm (**4**).

**Holotype**: ♀ (Fig. 1), body length 19.9 mm, cephalic plate 1.7 mm long, 1.9 mm broad, from the Mountain Yanzhi, Shandan County, Zhangye City, Gansu Province, 38°35'N, 101°41'E, 1395 m, 28 July 2007, leg. Z. Di, deposited in the School of Life Sciences, Hengshui University, Hengshui, China. **Paratypes**: 4 ♀♀, 4 ♂♂, same data as holotype.

###### Etymology.

the specific name *luculentus* refers to the moderately transparent protuberance on the dorsal terminal part of the second article of the female gonopods.

###### Diagnosis.

*Hessebius* with body length 15.8–19.9 mm, antennae composed of 20 articles; 9–10 ocelli on each side, arranged in 3 irregular rows, terminal ocellus comparatively large; Tömösváry’s organ smaller than the adjacent ocelli; 2+2 coxosternal teeth; porodonts moderately thick, posterolateral to the lateralmost tooth; posterior angles of all tergites without triangular projections; tarsal articulation well defined on legs 1–15; coxal pores 3–6, oval to round, arranged in one row; female gonopods with 2+2 moderately large, coniform spurs; dorsal terminal claw of the third article of the female gonopods simple, but with a small dentation in base; male gonopods short and small, with 3–4 long setae on the terminal segment.

###### Description.

body length: 15.8–19.9 mm, cephalic plate 1.4–1.7 mm long, 1.5–1.9 mm wide; colour: antennal articles yellow-brown; cephalic plate and tergites yellow-brown with a reddish hue, edge of tergites lighter; pleural region and all sternites pale yellow-brown; all legs pale yellow-brown with greyish hue; distal part of forcipules brownish black, basal and proximal parts of forcipules, forcipular coxosternite and TT 14 and 15 darker.

*Antennae*: 20+20 articles, one specimen with 20+22 articles; basal article slightly longer than wide, the second one markedly longer than wide, following articles gradually shortening, distal article up to 4.0 times as long as wide; abundant setae on the antennal surface, less so on the basal articles, gradually increase in density of setae to about sixth article, then more or less constant.

*Cephalic plate* smooth, convex, longer than wide; tiny setae emerging from pores scattered very sparsely over the whole surface; frontal marginal ridge with shallow anterior median furrow; short to long setae scattered along the marginal ridge of the cephalic plate; lateral marginal ridge discontinuous, posterior margin continuous, straight (Fig. 1).

Nine–ten oval to rounded *ocelli* on each side (Fig. 2), most of them rounded, domed, translucent, usually darkly pigmented, situated in three irregular rows; the posterior ones comparatively large; the adjoining ventral ocelli slightly smaller and the adjoining dorsal ones slightly larger.

*Tömösváry’s organ* situated at the anterolateral margin of the cephalic plate, moderately smaller than the adjoining ocelli and lying well apart from them (Fig. 2–To).

*Coxosternite* subtrapezoidal (Fig. 3), anterior margin narrow, lateral margins slightly longer than medial margins; median diastema moderately shallow, broad V-shaped; anterior margin with 2+2 subtriangular small sharp teeth; porodonts slender, lying posterolateral to and separated from the lateralmost tooth (Fig. 3), with slight bulge at base; scattered short setae on the ventral side of coxosternite, longer setae near the dental margin, more longer setae near the porodonts.

All *tergites* smooth, without wrinkles, dorsum slightly convex, tiny setae emerging from pores scattered sparsely over the entire surface, near the margin with few long setae; T 1 narrower posterolaterally than anterolaterally, generally trapezoidal, narrower than the cephalic plate and T 3, obviously longer than T 3, the cephalic plate slightly wider than T 3. Lateral marginal ridges of all tergites continuous. Posterior marginal ridges of TT 1 and 3 straight, continuous; posterior marginal ridges of TT 5, 7 and 8 slightly concave, discontinuous; posterior marginal ridges of TT 10, 12 and 14 concave, discontinuous. Posterior angles of tergites generally rounded, without triangular projections. Miniscule setae scattered sparsely over the surface, more numerous setae on anterior and posterior angles of each tergite, with 2–5 long setae on anterior angles and posterior angles of each tergite.

Posterior side of *sternites* narrower than anterior, generally trapezoidal, smooth; setae emerging from sparsely scattered pores on the surface and lateral margin, few long setae; a pair of longer setae approximately symmetrical on the surface of the anterior part of each sternite; 1–2 long setae on the surfaces both of the middle part and posterior part of each sternite.

*Legs* robust, tarsal articulation defined on legs 1–15, tarsus 1 longer than tarsus 2. All legs with fairly long curved claws. Legs 1–14 with anterior and posterior accessory spurs; anterior accessory spurs moderately long and slender, forming a moderately small angle with the claw, posterior accessory spurs slightly more robust, forming a comparatively large angle with the claw; leg 15 lacking accessory spurs; short to long setae sparsely scattered over the surface of prefemur, femur and tibia of legs 1–13, more setae on the tarsus, thicker setae scattered evenly over the tarsal surface, one row of thicker setae regularly arranged on the medial ventral side of tibia of legs 1–13, with setae significantly reduced on legs 14 and 15, no thicker setae regularly arranged in one row on the medial ventral side of tibia; legs 14 and 15 slightly thicker than the anterior pairs in the both female and male, especially in male; tarsus 1 5.1–5.3 times as long as wide. Leg plectrotaxy as in table 1.

**Table 1.** Leg plectrotaxy of *Hessebius luculentus* sp. n.

**Legs**	**Ventral**					**Dorsal**				
	C	Tr	P	F	Ti	C	Tr	P	F	Ti
1			mp	amp	am			ap	ap	a
2–8			mp	amp	am			ap	ap	ap
9			mp	amp	am			a(m)p	ap	ap
10–11			mp	amp	am			amp	ap	ap
12			mp	amp	am	(m)		amp	ap	ap
13		(m)	amp	amp	am	m		amp	ap	ap
14		m	amp	amp	am	m		amp	p	p
15		m	amp	am	a	m		amp	p	

Letters in brackets indicate variable spines.

*Coxal pores* 3–6, round, variable in size, arranged in a row; usually 4(5)6(5)6(5)6(5) in males and 3(4)554(4) in females. Coxal pore field set in a relatively shallow groove, the coxal pore-field fringe with prominence. Prominence with short to moderately long setae sparsely scattered over the surface.

**Female** S 15 with anterior margin broader than posterior, generally trapezoidal, posteromedially straight. Short to long sparse setae evenly scattered on surface. Surface of the lateral sternal margin of genital segment well chitinized, posterior margin of genital sternite deeply concave between condyles of gonopods, except for a small, median tongue-shape bulge. Relatively long setae scattered over ventral surface of the genital segment, few setae near S 15. Gonopods: first article fairly broad, bearing many short to moderately long setae about evenly scattered; with 2+2 moderately long and slender, coniform spurs, inner spur slightly smaller than the outer (Fig. 5); with 6 robust spines arranged in one irregular row dorsally on the posterior part of the external margin. Second article with approximately ten long setae, arranged in two irregular rows, with nine robust spines lying dorsally on the posterior part of the external margin, 6 of them arranged in an irregular longitudinal row, three of them arranged in an irregular transversal row; the dorsal terminal part extending backwards and forming a moderately transparent protuberance. Third article with 2–3 long setae ventrally, and two short, robust spines lying dorsally on the posterior part of the external margin (Fig. 6). Third article with a simple apical claw, and with a very small subtriangular denticle on inner margin (Fig. 7).

**Male** S 15 posterior margin narrower than anterior, posteromedially straight; density of setae on the surface of SS 13 and 14 in the male significantly increased, the S 15 is more significant, and the posterior more than the anterior; sternite of genital segment obviously smaller than the female, usually well sclerotized; posterior margin deeply concave between the gonopods, without medial bulge. Long setae scattered on the ventral surface of the genital segment, fewer setae near S 15, fringed with longer setae along the posterior margin; more than the female. Gonopods short, appearing as a small finger-shaped bulge, with 3–4 long setae, apically slightly sclerotized (Fig. 8).

###### Remarks.

The new species resembles *H. jangtseanus* (Verhoeff, 1942) from Sichuan Province, Central China, in having 9–10 ocelli on each side of the cephalic plate, 3–6 coxal pores, 2+2 spurs on the first article of the female gonopods, leg pair 15 lacking accessory spurs; but can be easily distinguished from *H. jangtseanus* by Tömösváry’s organ moderately smaller than the adjoining ocelli versus slightly larger than the adjoining ocelli or as large as the closest ocelli in *H. jangtseanus*; the dorsal end on the second article of the female gonopods forming a moderately transparent short protuberance instead of forming a long terminal spur pointing backwards as in *H. jangtseanus*; 15^th^ accessory spur absent versus present in *H. jangtseanus*; and both the 14^th^ and 15^th^ legs’ dorsal plectrotaxy obviously different: 10311 on legs 14 and 10310 on legs 15 compared to 10322 on legs 14 and 10320 on legs 15 in *H. jangtseanus*.

###### Habitat.

*Larix* forest at about 1400 m above sea level, in moderately moist habitats under roadside stones and litter of the forest floor.

To assist in the identification of the Chinese species of *Hessebius*, the following main morphological characters (table 2) and key to the known Chinese species of the genus based on adult specimens is presented.

**Table 2.** Main morphological characters of the known Chinese species of genus *Hessebius* Verhoeff, 1941 based on adult specimens.

	*luculentus* sp. n.	*jangtseanus*	*longispinipes*	*multiforaminis*
Sources	this paper	Verhoeff 1942; Pei et al. 2010	Ma et al. 2009	Pei et al. 2010
Distribution	China SW (Gansu)	China SE (Sichuan)	China NW (Xinjiang Uygur)	China SW (Tibet)
Body length (mm)	15.8–19.9	6.9 – 14.0	10.7 – 12.6	18.9 – 22.9
Number of antennal articles	20+20	19+19 – 22+22, commonly 20+20	17+17 – 19+19, commonly 18+18	20+20
Number, arrangement and shape of the ocelli	9 – 10 in 3 irregular rows, oval to rounded, commonly rounded	9–12, oval to round, in 3 rows	1+3, 2, oval to rounded, commonly oval, in 2 irregular rows	13 (1+5,4,3)–15 oval to rounded, commonly rounded, in 3 irregular rows
Posterior ocellus	oval, small	oval to round, comparatively large	oval, larger than the seriate ocelli	bigger than the seriate ocelli
Seriate ocelli	the adjoining ventral ocelli slightly smaller, the adjoining dorsal ocelli slightly larger	moderately small, approximately equal in size	moderately small, approximately equal in size; posterosuperior ocellus of the same size or slightly larger than other seriate ocelli	moderately small, approximately equal in size except the posterosuperior ocellus comparatively larger than other seriate ocelli
Tömösváry’s organ	moderately smaller than the adjoining ocelli	moderately large, rounded, slightly bigger than the adjoining ocelli or as big as the closest ocelli	moderately small, nearly rounded, about same size as the adjoining ocelli	very small, rounded; comparatively close to the adjoining ocelli
Number and arrangement of coxosternal teeth	2+2, triangular	2+2, moderately sharp	2+2, triangular, terminal part of each tooth slightly blunt	2+2, comparatively sharp, terminal part of each tooth approximately blunt
Porodont	porodonts slender, lying posterolateral to the lateralmost, apart from the tooth	moderately thickset	moderately stout, just posterolateral to the lateral tooth, without bulge near the base	moderately stout, just posterolateral and moderately far from the lateral tooth, without bulge near their base
Tergites	smooth	smooth	moderately smooth, without wrinkles	moderately rough, with some wrinkles
Number of coxal pores	usually 4(5)6(5)6(5)6(5) in males and 3(4)554(4) in females.	4454 (Verhoeff, 1942); or 4–6	2–5: usually 2343 (male); 3444, 2444 (female)	4–7: usually 6766, 7777, 5666, 5666 (male); 5676, 5564, 4554, 66(7)6(5) (female)
Shape of coxal pores	round	ovate to round	round or slightly ovate, small to moderately large	round or slightly ovate, small to moderately large
Tarsus 1–tarsus 2 articulation on legs 1–13	well–defined	not well–defined	not well–defined	not well–defined
Male 14^th^ leg	slightly thicker than 1–13 legs	thicker than 1–13 legs	moderately thicker and stronger than 1–13 legs, more thicker and stronger than female	markedly thicker and stronger than 1–13 legs,
Male 15^th^ leg	slightly thicker than 1–13 legs	thicker than 1–13 legs	moderately thicker and stronger than 1–13 legs; more thicker and stronger than in female	markedly thicker and stronger than in 1–13 legs
Dorsal sulci on male 14^th^ and 15^th^ legs	absent	absent	a comparatively obvious dorsal furrow on the tibia of legs 14 and 15	two comparatively obvious shallow dorsal furrow on the tibia of legs 14 and 15
DaC spine	on (12^th^) 13^th^–15^th^ legs	on 13^th^–15^th^ legs	on 9^th^–15^th^ legs	on 12^th^–15^th^ legs
14^th^ accessory spur	present on both anterior and posterior side of the claw	present on both anterior and posterior side of the claw	absent	present on both anterior and posterior side of the claw
15^th^ accessory spur	absent	present	absent	absent
Number and shape of spurs on female gonopods	2+2 moderately long and slender, bullet-shaped	2+2, thick, bullet–shaped	2+2, moderately long, bullet–shaped, the inner slightly smaller and more anterior than the outer	2+2, moderately long, bullet–shaped
Shape of dorsal terminal thorn on 2^nd^ article of female gonopods	extending backwards and forming a moderately transparent protuberance	moderately feeble long terminal spur pointing backwards	strongly extending backwards and forming a thick and long terminal thorn	strongly extending backwards and forming a thick terminal protuberance
Apical claw of female gonopods (and lateral denticles)	simple, with a very small subtriangular denticle on inner margin	simple, only with one small ventral triangular denticle	simple, slender and sharp, with moderately small protuberance on both ventral and dorsal sides, the dorsal one more anterior; usually 3 moderately long setae	simple and broad
Male gonopods	short, appearing as a small fingered bulge, with 3–4 long setae, apically slightly sclerotized	short and small, only a small hemispherical bulge, with 1–2 long setae on the surface, tip slightly sclerotised	short and small, only a small hemispherical bulge, with 2 long setae on surface, terminal slightly sclerotised	short and small, only a small hemispherical bulge, with 6–8 long setae on surface, terminal slightly sclerotised

#### Key to the Chinese species of genus *Hessebius*

**Table:** 

1	Four ocelli on each side of the cephalic plate, the dorsal terminal part of second article of the female gonopods strongly extending backwards and forming a thick and long terminal thorn	***H. longispinipes***
–	At least 9 ocelli on each side of cephalic plate, the dorsal terminal part of second article of the female gonopods not strongly extending backwards nor forming a thick and long terminal thorn	**2**
2	At least 13 ocelli on each side of the cephalic plate	***H. multiforaminis***
–	At most 12 ocelli on each side of the cephalic plate	**3**
3	Dorsal plectrotaxy on leg 15 is 10320	***H. jangtseanus***
–	Dorsal plectrotaxy on leg 15 is 10310	***H. luculentus* sp. n.**

## Supplementary Material

XML Treatment for Hessebius luculentus
